# Association between BMP4 rs17563 Polymorphism and NSCL/P Risk: A Meta-Analysis

**DOI:** 10.1155/2015/763090

**Published:** 2015-01-12

**Authors:** Yuan-Yuan Hu, Chuan-Qi Qin, Mo-Hong Deng, Yu-Ming Niu, Xing Long

**Affiliations:** ^1^Department of Oral and Maxillofacial Surgery, The State Key Laboratory Breeding Base of Basic Science of Stomatology and Key Laboratory of Oral Biomedicine, Ministry of Education, School and Hospital of Stomatology, Wuhan University, Luoyu Road 237, Wuhan 430079, China; ^2^Department of Stomatology and Evidence-Based Medicine Center, Taihe Hospital, Hubei University of Medicine, 32 South Renmin Road, Shiyan 442000, China

## Abstract

*Objective.* To investigate the association between bone morphogenetic protein 4 (BMP4) rs17563 polymorphism and nonsyndromic cleft lip with or without palate (NSCL/P) risk.* Methods.* Four online databases were researched and the related publications were collected. Odds ratio (OR) with 95% confidence interval (CI) was applied to assess the relationship; publication bias, metaregression, and sensitivity analysis were conducted to guarantee the strength of results.* Results.* Six published case-control studies were collected. Overall, no significant association between BMP4 rs17563 polymorphism and NSCL/P risk was found. It was notable that significant susceptibility on different ethnicity was observed in the stratified analysis. For Chinese population, the BMP4 rs17563 polymorphism was a significantly increased risk for NSCL/P (C versus T: OR = 1.52, 95% CI = 1.28–1.82, *P* < 0.01, *I* ^2^ = 0%; CC versus TT: OR = 2.58, 95% CI = 1.74–3.82, *P* < 0.01, *I* ^2^ = 0%; TC + CC versus TT: OR = 1.45, 95% CI = 1.14–1.84, *P* < 0.01, *I* ^2^ = 0%; CC versus TT + TC: OR=2.46, 95% CI = 1.46–4.14, *P* < 0.01, *I* ^2^ = 47.0%). On the contrary, significantly protective effects were found in Brazilian population (C versus T: OR = 0.69, 95% CI = 0.50–0.96, *P* = 0.03, *I* ^2^ = 68.5%; TC versus TT: OR = 0.52, 95% CI = 0.40–0.68, *P* < 0.01, *I* ^2^ = 0%; TC + CC versus TT: OR = 0.52, 95% CI = 0.35–0.78, *P* < 0.010, *I* ^2^ = 54.4%).* Conclusion*. This meta-analysis indicated that BMP4 rs17563 polymorphism could play a different role during the development of NSCL/P based on ethnicity diversity.

## 1. Introduction

Nonsyndromic cleft lip with or without palate (NSCL/P) is among the most common birth defects. NSCL/P occurs in approximately 1/700 to 1/1,000 live births in different areas and ethnicities worldwide [1]. Among Chinese newborns, the prevalence of NSCL/P has been reported to reach 1.42/1000. NSCL/P patients are subjected to long-term medical costs and mental disability, thereby causing a substantive burden to their families and the society [2].

Epidemiological studies have indicated that NSCL/P development is due to the interaction between genetic and environmental factors. Genetic mutation, unhealthy habits (cigarette smoking and alcohol drinking), vitamin deficiency, and pollution increase the risk of NSCL/P. For genetic factors, approximately 20 genes are involved in the etiology of NSCL/P, including TGF-beta, MTHFR, AXIN2, IRF6, and MSX1 [3–6].

Animal models showed that the bone morphogenetic protein 4 gene (BMP4) is among the potential candidate genes for NSCL/P [7]. Liu et al. reported that all embryos of BMP4 knockout mouse model had bilateral cleft lip at the 12th embryonic day, thereby indicating that the BMP signal pathway is critical for cell proliferation and fusion during the early stage of oral and maxillofacial construction [8]. Moreover, experimental research showed that BMP4 is expressed at the fusional area of mice facial processes, which indicates that BMP4 could serve an important function in regulating lip fusion during midfacial morphogenesis [9].

BMP4 is located at chromosome 14q22-23, contains a cDNA of 1227b with four exons, and encodes the BMP4 protein [10]. One of the most functional single-nucleotide polymorphisms (SNPs) of BMP4 gene, rs17563, is currently a research focus. This SNP showed the change from T to C at 538 nucleotide position (538T/C), which is a promising candidate SNP locus associated with positive risk for NSCL/P development.

In 2008, the first molecular epidemiological study was reported by Lin et al. [11]. Significantly increased risk was found between BMP4 rs17563 and NSCL/P. Many studies have been published on this topic, but the results were inconsistent. Therefore, we conducted this meta-analysis to clarify the association between BMP4 rs17563 and NSCL/P.

## 2. Materials and Methods

### 2.1. Search Strategy

Four electronic databases (PubMed, Embase, CNKI, and Wanfang) were searched to collect related studies. Only studies in English and Chinese were selected. The research terms included “bone morphogenetic protein 4,” “BMP4,” “nonsyndromic cleft lip with or without palate,” “cleft,” “palate,” and “polymorphism.” Furthermore, the bibliographies of some review articles were retrospectively used as complements to identify potential studies.

### 2.2. Inclusion and Exclusion Criteria

All selected studies met the following inclusion criteria: (1) case-control or cohort design study on NSCL/P; (2) focus on BMP4 rs17563 polymorphism; (3) sufficient genotype frequency to estimate the odds ratio (OR) and 95% confidence interval (CI); and (4) being published in English or Chinese. Exclusion criteria include (1) review articles; (2) case reports; (3) preliminary results that do not include BMP4 rs17563 gene polymorphism or outcome; and (4) animal models researchers. The largest or most recently published studies were selected when similar or overlapping data are present.

### 2.3. Data Extraction

Data, that is, senior author's name, year of publication, sources of controls, country where the study was conducted, ethnicity of participants (such as Asian or Caucasian), genotyping method, and number of different genotypes of cases and controls, were extracted from relevant studies collected. The abovementioned data was independently completed by two reviewers (Hu and Qin). A third reviewer (Deng) examined all discrepancies in the analyses for consistency. Hardy-Weinberg equilibrium (HWE) was calculated based on the genotypes in the controls.

### 2.4. Statistical Analysis

Heterogeneity was assessed using Cochran's *Q* statistic and *I* ^2^ method. The strength of the association between the NSCL/P and BMP4 rs17563 polymorphism risk was assessed by crude ORs with 95% confidence intervals (CIs), which comprised the following models: allele contrast (C versus T); codominant (TC versus TT and CC versus TT); dominant (TC + CC versus TT); and recessive (CC versus TT + TC). If the *P* value < 0.10 or *I* ^2^ was greater than 40%, the summary OR estimation was calculated with a random-effect model (DerSimonian and Laird method). Otherwise, the fixed-effect model (Mantel-Haenszel method) was adopted. Cumulative meta-analyses and sensitivity analysis were conducted to evaluate the stability of the results for each model. Potential publication bias was evaluated by Egger's linear regression and Begg's funnel plots. To adjust for multiple comparisons, we applied Bonferroni method with R software. Statistical analysis was performed using STATA version 11.0 (Stata Corporation, College Station, TX, USA) and R software (version 3.1.1). All *P* values were two-sided. *P* < 0.05 was considered significant.

## 3. Results

### 3.1. Study Characteristic

**Figure 1:**
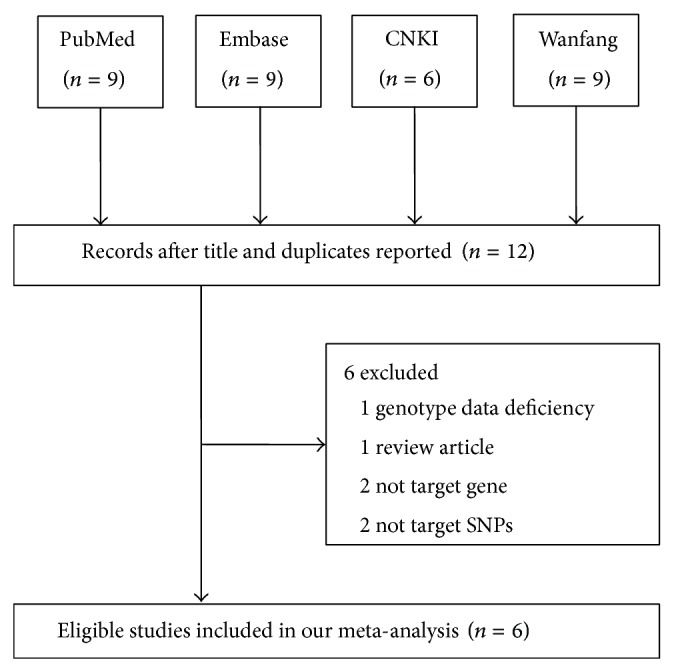
Flow diagram of the study selection process.

Thirty-three relevant studies were selected based on the search words. The flow chart of study selection is presented in Figure 1. After screening the titles and duplicated reports, 21 of the publications were excluded. Abstracts and full texts of the remaining 12 articles were reviewed, and six studies were subsequently excluded. Finally, six publications with eligible genotypes were selected. These studies included 1,040 cases and 1,260 controls [12–17] and were subjected to meta-analysis. No study deviated from HWE. Also, the minor allele frequency of the C allele ranged from 0.27 to 0.52. The characteristics of included studies are summarized in Table 1.

### 3.2. Meta-Analysis

**Figure 2:**
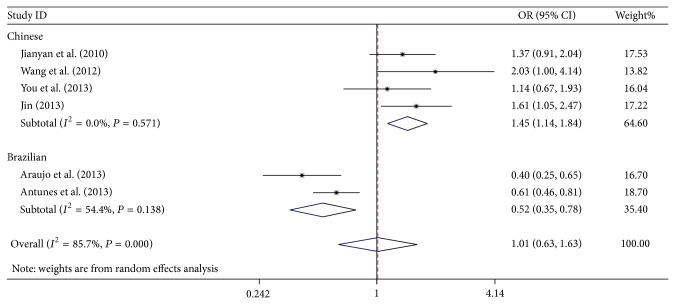
OR and 95% CIs for the association between BMP4 rs17563 polymorphism and NSCL/P risk in TC + CC versus TT model.

In general, no significant association was found between NSCL/P and BMP4 rs17563 polymorphism in the meta-analysis of five genotype models. Interestingly, the two ethnicities (Chinese Han and Brazilian) showed different susceptibilities to NSCL/P development. For the Chinese population, BMP4 rs17563 polymorphism possibly contributed to a significant increase in NSCL/P risk (for allele contrast, C versus T: OR = 1.52, 95% CI = 1.28–1.82, *P* < 0.01, *I* ^2^ = 0%; codominant model, CC versus TT: OR = 2.58, 95% CI = 1.74–3.82, *P* < 0.01, *I* ^2^ = 0%; dominant model, TC + CC versus TT: OR = 1.45, 95% CI = 1.14–1.84, *P* < 0.01, *I* ^2^ = 0%, Figure 2; and recessive model, CC versus TT + TC: OR = 2.46, 95% CI = 1.46–4.14, *P* < 0.01, *I* ^2^ = 47.0%). By contrast, significantly protective effects were found in the Brazilian population (for allele contrast, C versus T: OR = 0.69, 95% CI = 0.50–0.96, *P* = 0.03, *I* ^2^ = 68.5%; for codominant model, TC versus TT: OR = 0.52, 95% CI = 0.40–0.68, *P* < 0.01, *I* ^2^ = 0%; and for dominant model, TC + CC versus TT: OR = 0.52, 95% CI = 0.35–0.78, *P* < 0.010, *I* ^2^ = 54.4%, Figure 2). Furthermore, no substantial changes were found about the adjusted *P* value in the five genetic models of overall and subgroup analysis; it also showed that the statistical results were reliable. Heterogeneity was found in all genotype models. Metaregression was conducted and the results indicated that ethnicity could explain the *τ* ^2^ values in the genetic models (for C versus T, *P* = 0.006; for TC versus TT, *P* = 0.033; for CC versus TT, *P* = 0.019; for TC + CC versus TT, *P* = 0.006; for CC versus TT + TC, *P* = 0.058). The subsequently stratified analysis indicated that heterogeneity was absent in the Asian population (Table 2).

### 3.3. Sensitivity Analysis

**Figure 3:**
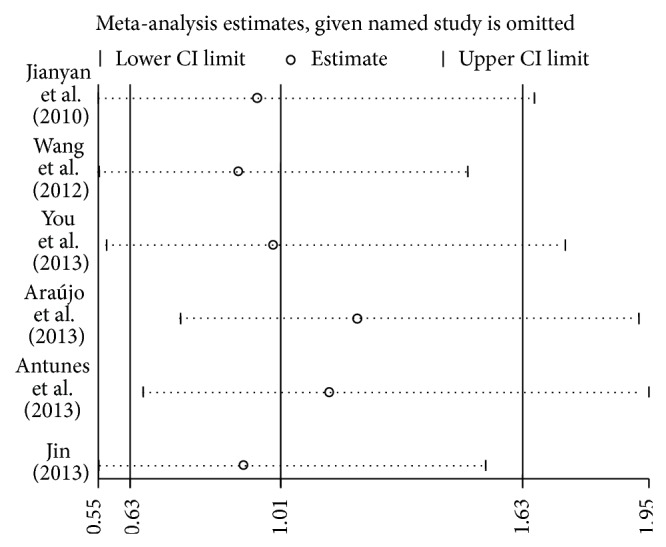
Sensitivity analysis through deleting each study to reflect the influence of the individual dataset to the pooled ORs in TC + CC versus TT model of BMP4 rs17563 polymorphism.

Each study in the meta-analysis was deleted one by one to reflect the effect of an individual dataset to the pooled ORs. The results were consistent (Figure 3 for the dominant model) in the genetic model studies, indicating that our results are statistically robust.

### 3.4. Publication Bias

**Figure 4:**
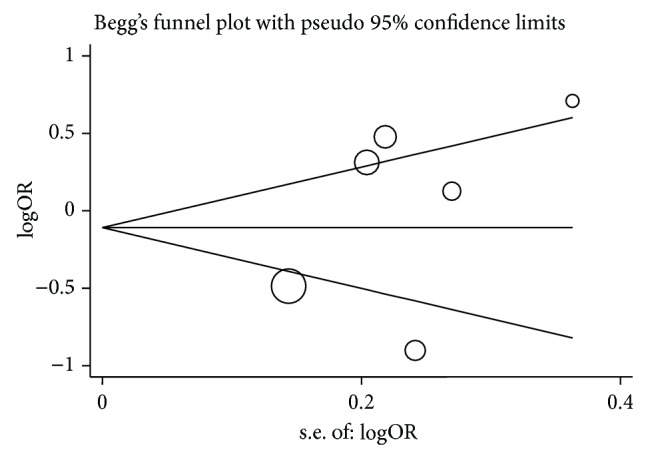
Funnel plot analysis to detect publication bias for TC + CC versus TT model of BMP4 rs17563 polymorphism. Each point represents a separate study.

Funnel plot and Egger's test were performed to estimate the publication bias of the studies. The shapes of the funnel plots in all genetic models failed to reveal any asymmetrical evidence.  Figure 4 shows the shapes of the funnel plots of the dominant model, which was used in the studies to examine the populations. The result was further supported after data analysis using Egger's test. No significant publication bias was found in this meta-analysis (*P* = 0.208 for C versus T; *P* = 0.394 for TC versus TT; *P* = 0.213 for CC versus TT; *P* = 0.320 for TC + CC versus TT; *P* = 0.256 for CC versus TT + TC).

## 4. Discussion

NSCL/P is the most common congenital malformation of the oral and maxillofacial region and is caused by a combination of genetic and environmental factors. Molecular research showed that many signaling pathways participate in the connection and fusion of the epithelium during the development of lip and palate. MTHFR, IRF6, MSX1, and TGF are proven risk factors for NSCL/P.

Recently, a number of studies focused on the mechanism of BMP4 during NSCL/P development. Immunohistochemical studies show that BMP4 could be expressed in the edge epithelium at the fusion of the maxillary prominence, which forms the lateral parts of the upper lip and the secondary palate [9, 18]. In an experiment using BMP4 gene knockout mice, nearly all embryos exhibited cleft lip [8]. All studies indicate that the BMP4 gene is a promising candidate for NSCL/P.

BMP4, located at chromosome 14q22-23, is a member of the BMP family and belongs to the transforming growth factor-beta (TGF-*β*) superfamily. In 1999, Mangino first reported a promising candidate SNP in the human BMP4 gene, in which the amino acid valine was replaced by alanine in exon 4 (Val152Ala) [19]. In 2008, Lin et al. [11] were the first to report that the CC genotype was associated with a significantly increased risk of NSCL/P compared with the TT genotype (ORs = 2.83, 95% CI: 1.46–5.47, and *P* = 0.002). The same results were also found in the overlapping and expanding data of Jianyan and colleagues [17]. However, results from association analysis have not been consistent. Interestingly, all published reports focused on Chinese and Brazilian populations. In a study of a Northeast China population in 2013, Jin found that the TC genotype elevated the risk for NSCL/P compared with the TT genotype (OR = 1.60, 95% CI: 1.02–2.52, and *P* = 0.042) [16]. Furthermore, other studies provided further evidence of the significant association between BMP4 rs17563 C mutation and NSCL/P risk [12, 13]. Two studies revealed conflicting conclusions in a Brazilian population. Araújo et al. [14] found that the BMP4 rs17563 C allele may have a protective effect on NSCL/P occurrence (OR = 0.40, 95% CI = 0.25–0.65, and *P* = 0.00018). Antunes et al. [15] suggested that BMP4 rs17563 polymorphism could be a clinically important protective factor in NSCL/P etiology. Ethnicity differences may be the most critical factor affecting diverse NSCL/P susceptibility among the Chinese and Brazilian populations.

In our meta-analysis, the nucleotide mutation form T to C of BMP4 rs17563 will increase the risk of developing NSCL/P in Chinese population; on the contrary, the protective effects of T allele were found in Brazilian population. The different or adverse results indicated that the BMP4 rs17563 polymorphism maybe play a different role during the development of NSCL/P in different ethnicity.

This meta-analysis has several limitations. First, all results were from unadjusted estimates and lacked the original data from the included studies. The relationship between gene-environment interactions and NSCL/P development was not explained. Second, because of the small sample size and the limited number of publications, the exact association between BMP4 rs17563 C mutation and NSCL/P risk was not revealed. Third, only studies on Chinese and Brazilian populations were included in our meta-analysis, thereby suggesting the presence of ethnicity bias.

In summary, our meta-analysis primarily indicated that BMP4 rs17563 polymorphism may serve an important function in the development of NSCL/P based on ethnicity differences. Future case-control studies with large sample sizes are required to investigate the potential mechanism between BMP4 rs17563 polymorphism and NSCL/P.

## Figures and Tables

**Table 1:** Characteristics of case-control studies on BMP4 rs17563 T>C polymorphism and NSCL/P risk included in the meta-analysis.

First author	Year	Country	Racial/descent	Source of controls	Case	Control	Genotype distribution						*P* for HWE^a^	Genotyping type	MAF
							Case			Control					
							TT	TC	CC	TT	TC	CC			
Jianyan et al. [17]	2010	Chinese	Asian	Hospital control	200	200	74	90	36	89	94	17	0.258	PCR-RFLP	0.32
Wang [12]	2012	Chinese	Asian	Hospital control	65	65	21	32	12	32	28	5	0.740	PCR-RFLP	0.29
You [13]	2013	Chinese	Asian	Population control	116	123	40	40	36	46	66	11	0.063	PCR-RFLP	0.36
Araújo [14]	2012	Brazilian	Mixed	Population control	123	246	49	53	21	52	130	64	0.351	PCR-RFLP	0.52
Antunes [15]	2013	Brazilian	Mixed	Hospital control	382	436	176	147	59	150	224	62	0.137	Taqman	0.40
Jin [16]	2013	Chinese	Asian	Hospital control	154	190	66	70	18	104	69	17	0.264	PCR-RFLP	0.27

^a^HWE in control.

MAF: minor allele frequency.

**Table 2:** Summary ORs and 95% CI of BMP4 rs17563 T>C polymorphism and NSCL/P risk.

	*N* ^*^	C vs. T					TC vs. TT					CC vs. TT					TC + CC vs. TT					CC vs. TT + TC				
		OR	95% CI	*P*	*I* ^2^ (%)^a^	Bon	OR	95% CI	*P*	*I* ^2^ (%)^a^	Bon	OR	95% CI	*P*	*I* ^2^ (%)^a^	Bon	OR	95% CI	*P*	*I* ^2^ (%)^a^	Bon	OR	95% CI	*P*	*I* ^2^ (%)^a^	Bon
Total	6	1.17	0.81–1.68	0.40	87.6	1	0.88	0.56–1.38	0.58	81.7	1	1.51	0.72–3.15	0.27	85.9	1	1.01	0.63–1.63	0.96	85.7	1	1.63	0.91–2.90	0.10	80.3	0.49
Ethnicity																										
Chinese	4	**1.52**	**1.28–1.82**	**<0.01**	**0**	**<0.01**	1.21	0.83–1.76	0.32	49.2	1	**2.58**	**1.74–3.82**	**<0.01**	**0**	**<0.01**	**1.45**	**1.14–1.84**	**<0.01**	**0**	**0.02**	**2.46**	**1.46–4.14**	**<0.01**	**47.0**	**<0.01**
Brazilian	2	**0.69**	**0.50–0.96**	**0.03**	**68.5**	0.14	**0.52**	**0.40–0.68**	**<0.01**	**0**	**<0.01**	0.55	0.24–1.26	0.16	79.3	0.78	**0.52**	**0.35–0.78**	**<0.01**	54.4	**<0.01**	0.83	0.45–1.54	0.55	70.7	1

^*^Numbers of comparisons.

^a^The variation in OR attributable to heterogeneity.

Bon: *P* value with Bonferroni testing of multiple comparisons.

## References

[1] Yuan Q., Blanton S. H., Hecht J. T. (2011). Genetic causes of nonsyndromic cleft lip with or without cleft palate. *Advances in Oto-Rhino-Laryngology*.

[2] Dai L., Zhu J., Mao M. (2010). Time trends in oral clefts in Chinese newborns: data from the Chinese national birth defects monitoring network. *Birth Defects Research Part A: Clinical and Molecular Teratology*.

[3] Lu X.-C., Yu W., Tao Y. (2014). Contribution of transforming growth factor *α* polymorphisms to nonsyndromic orofacial clefts: a HuGE review and meta-analysis. *American Journal of Epidemiology*.

[4] Estandia-Ortega B., Velázquez-Aragón J. A., Alcántara-Ortigoza M. A., Reyna-Fabian M. E., Villagómez-Martínez S., González-del Angel A. (2014). 5,10-Methylenetetrahydrofolate reductase single nucleotide polymorphisms and gene-environment interaction analysis in non-syndromic cleft lip/palate. *European Journal of Oral Sciences*.

[5] Letra A., Bjork B., Cooper M. E. (2012). Association of *AXIN2* with non-syndromic oral clefts in multiple populations. *Journal of Dental Research*.

[6] Song T., Wu D., Wang Y., Li H., Yin N., Zhao Z. (2013). SNPs and interaction analyses of IRF6, MSX1 and PAX9 genes in patients with non-syndromic cleft lip with or without palate. *Molecular Medicine Reports*.

[7] Lu H., Jin Y., Tipoe G. L. (2000). Alteration in the expression of bone morphogenetic protein-2,3,4,5 mRNA during pathogenesis of cleft palate in BALB/c mice. *Archives of Oral Biology*.

[8] Liu W., Sun X., Braut A. (2005). Distinct functions for Bmp signaling in lip and palate fusion in mice. *Development*.

[9] Gong S.-G., Guo C. (2003). *Bmp4* gene is expressed at the putative site of fusion in the midfacial region. *Differentiation*.

[10] van Den Wijngaard A., Weghuis D. O., Boersma C. J. C., van Zoelen E. J. J., Van Kessel A. G., Olijve W. (1995). Fine mapping of the human bone morphogenetic protein-4 gene (BMP4) to chromosome 14q22-q23 by in situ hybridization. *Genomics*.

[11] Lin J.-Y., Chen Y.-J., Huang Y.-L. (2008). Association of bone morphogenetic protein 4 gene polymorphisms with nonsyndromic cleft lip with or without cleft palate in Chinese children. *DNA and Cell Biology*.

[12] Wang H., Zhou X., Cui Y., Liu J., Wang W. (2012). Relationship between nonsyndromic cleft lip with or without cleft palate (NSCL/P) and genentic polymorphisms of BMP4. *Jiangsu Medical Journal*.

[13] You L., Jiao X., Zhang B., Yin X., Tan C., Hao Y. (2013). Association of BMP4 polymorphisms with non-syndromic cleft lip with or without cleft palate in a northern Chinese population. *Beijing Journal of Stomatology*.

[14] Araújo T. K., Simioni M., Félix T. M. (2012). Preliminary analysis of the nonsynonymous polymorphism rs17563 in *BMP4* gene in Brazilian population suggests protection for nonsyndromic cleft lip and palate. *Plastic Surgery International*.

[15] Antunes L. S., Küchler E. C., Tannure P. N. (2013). *BMP4* polymorphism is associated with nonsyndromic oral cleft in a Brazilian population. *The Cleft Palate-Craniofacial Journal*.

[16] Jin S. (2013). *Investigation on the relation of NSCL/P and BMP4 gene T538C mutation in Northeast China [M.S. thesis]*.

[17] Jianyan L., Zeqiang G., Yongjuan C., Kaihong D., Bing D., Rongsheng L. (2010). Analysis of interactions between genetic variants of BMP4 and environmental factors with nonsyndromic cleft lip with or without cleft palate susceptibility. *International Journal of Oral and Maxillofacial Surgery*.

[18] Tapadia M. D., Cordero D. R., Helms J. A. (2005). It's all in your head: new insights into craniofacial development and deformation. *Journal of Anatomy*.

[19] Mangino M., Torrente I., De Luca A., Sanchez O., Dallapiccola B., Novelli G. (1999). A single-nucleotide polymorphism in the human bone morphogenetic protein-4 (BMP 4) gene. *Journal of Human Genetics*.

